# Garcinol Encapsulated Ph-Sensitive Biodegradable Nanoparticles: A Novel Therapeutic Strategy for the Treatment of Inflammatory Bowel Disease

**DOI:** 10.3390/polym13060862

**Published:** 2021-03-11

**Authors:** Eden Mariam Jacob, Ankita Borah, Sindhu C. Pillai, D. Sakthi Kumar

**Affiliations:** Bio Nano Electronics Research Centre, Graduate School of Interdisciplinary New Science, Toyo University, 2100 Kujirai, Kawagoe, Saitama 350-8585, Japan; edenmjkoshy@gmail.com (E.M.J.); ankitaborah24@gmail.com (A.B.); sindhucpillai@hotmail.com (S.C.P.)

**Keywords:** inflammatory bowel disease, pH-sensitive nanoparticles, eudragit S100, garcinol, nuclear factor kappa light chain enhancer of activated B-cells

## Abstract

The emergence of pH-sensitive nanoscale particles is beneficial due to their ability to only release cargo in a colonic pH environment, which helps to directly target inflamed tissues in inflammatory bowel disease (IBD). Hence, we have designed the formulation of pH-sensitive biodegradable garcinol (GAR)-loaded poly (lactic–*co*–glycolic acid) (PLGA) coated with Eudragit^®^ S100 (ES100) (GAR-PLGA-ES100 nanoparticles (NPs)) for reducing inflammation caused by proinflammatory cytokines. The GAR-PLGA-ES100 NPs were prepared using a solvent evaporation technique and characterized for shape and surface morphology. An in vitro drug release study revealed the release of the drug specifically from NPs at the colonic pH of 7.4. The in vitro cytotoxicity of the GAR-PLGA-ES100 NPs was also evaluated and found to be highly biocompatible with CACO-2 cells. These NPs were able to reduce lactate dehydrogenase (LDH) and myeloperoxidase (MPO) activity. Inhibition of the expression of pro-inflammatory cytokine TNF-α , chemokine interleukin (IL)-8 and the nuclear factor kappa light chain enhancer of activated B-cells (NF-κB) was observed after GAR-PLGA-ES100 NPs treatment. Therefore, our results support the idea that GAR-PLGA-ES100 NPs show substantial improvement after the release of the drug, specifically in colonic pH targeting and reduction in the activation of inflammation that leads to IBD, suggesting that GAR-PLGA-ES100 NPs are promising candidates for oral delivery to colonic inflamed tissue.

## 1. Introduction

Inflammatory bowel disease (IBD) is classified into two types: Crohn’s disease (CD) and ulcerative colitis (UC) with unknown etiology [1]. The difference between CD and UC depends on the position, intestinal layer, and progression [2]. The most common symptoms of the disease include abdominal pain, diarrhea [3], rectal bleeding, and weight loss [4]. Although the disease’s etiology is not fully understood, various contributing factors such as altered genetic background, gut immunity, microbial dysbiosis, external environmental conditions including diet, and new lifestyle practices are involved in the pathogenesis of the disease [5]. During inflammation, intestinal epithelial cells (IEC) receive activating signals from pro-inflammatory cytokines, including tumor necrosis factor (TNF)-α, interleukin (IL)-1β, and IL-6 produced in the activated immune cells of the gut. One of the commonly identified genes is nucleotide-binding oligomerization domain 2/Caspase recruitment domain-containing protein 15 (NOD2/CARD15) [6], which induces the signaling of nuclear factor kappa B-cells (NF-κB) cascade and mitogen-activated protein kinase (MAPK)-dependent pathways when faced with bacterial peptidoglycans [7].

Over the last years, biological therapies, such as antibodies against TNF-α and various steroidal drugs, were implemented as a therapeutical strategy for treating IBD, depending on the severity of the patients [8]. Often, IBD patients become dependent on these conventional drug therapies for their whole lives, burdened with more severe complications and the additional toxic side effects associated with them [9]. Additionally, pellets, capsules or tablets that are designed for sustained drug release at the colon over a long period show reduced therapeutic efficacy and are only useful in a subset of IBD patients [10]. Nanomedicine therapy can overcome these challenges by delivering active ingredients to the site of inflammation in a controlled fashion and consequently contribute to lesser adverse effects [5]. One of the significant concerns involves the pH of the colon, which is often altered in IBD and can, in turn, affect other factors like enzymatic degradation, transit time, and colonic bacterial load [11]. Variations in the physiological and chemical features of the gastrointestinal tract (GIT) can lead to elevated inflammation. pH-controlled drug delivery systems utilize the alteration of pH in the GIT as a factor for the controlled release of the drug at the target site [12]. pH-sensitive polymers such as poly (methacrylic acid–*co*–methyl methacrylate) and Eudragit^®^ S100 (ES100) are designed to be released at the specific pH of the colon (pH ≥ 7) [13]. Interestingly, poly (lactic acid-co- glycolic acid) (PLGA) is a synthetic biodegradable polymer that has also been studied to attain a sustained drug release pattern for long-term therapeutic action [14]. Synthesis of NPs composed of PLGA and Eudragit FS30D were studied and only released cyclosporine at pH 7.4 in vitro due to the pH-sensitive nature of Eudragit FS30D and thereby sustained release property due to PLGA [15]. The development of nanoparticles using biodegradable materials that can improve pH-sensitive delivery of specific drugs can ensure the eradication of inflammation without injuring nearby healthy tissue [16].

LIVINOL^TM^ (garcinol (GAR)), a natural compound from the fruit *Garcinia indica*, has also been used in cooking and treatment for gastric disorders and skin irritation [17]. Various studies have claimed that GAR is a strong antioxidant, anti-inflammatory and anticancer agent [18]. The encapsulation of GAR in polymeric nanoparticles (NPs) for increasing its bioavailability is still new in the nanomedicine therapeutic field [19]. Previous studies have indicated that GAR was found to be more effective in inducing apoptosis than curcumin when treated on human leukemia HL-60 cells in a dose-dependent manner [20]. So far, very few studies have been conducted on GAR and IBD due to their anti-inflammatory effect; hence, we have designed a pH-sensitive biodegradable nanocarrier for GAR using PLGA coated with ES100 (GAR-PLGA-ES100 NPs). The synthesis of polymeric NPs involves various techniques that have been traditionally utilized, including solvent displacement, emulsification-solvent evaporation, nano precipitation, supercritical fluid technology, salting-out, and the dialysis method [21]. Different techniques have distinct advantages and disadvantages; key aspects of NP synthesis involve polymer concentration, uniformity of NP size, drug encapsulation efficiency, solvent type, surfactant concentration and a mixing device [22]. For the synthesis of GAR-PLGA-ES100 NPs, the method of solvent evaporation was chosen as it is one of the most widely anticipated techniques for loading hydrophobic drugs into NPs. GAR-PLGA-ES100 NPs were designed to load and deliver the drug for targeted delivery to the colon to treat IBD and inhibit inflammation by reducing NF-κB, proinflammatory cytokine TNF-α and chemokine interleukin (IL)-8 in CACO-2 cells.

## 2. Materials

PLGA (L: G, 50: 50, average Molecular weight (M_w_) 38,000–54,000) and polyvinyl alcohol (PVA, average (M_w_ 30,000–50,000), hydrolysis degree 87–89%) were procured from Sigma-Aldrich (St Louis, MO, USA). Eudragit^®^ S100 (ES100) was a gift from Evonik, (Essen, Germany), and LIVINOL^TM^ (GAR) was a gift from Sami Sabinsa Group, Sami Labs (Bengaluru, India). Phosphate buffered saline (PBS) was purchased from Gibco (Life technologies: Carlsbad, CA, USA), fetal bovine serum (FBS) was bought from Biowest (Riverside, MO, USA), 0.25% Trypsin-EDTA from Gibco Technologies (Waltham, MA, USA), and Dimethyl sulfoxide (DMSO) was purchased from Kanto Chemicals (Tokyo, Japan). Coumarin-6 was procured from Tokyo Chemical Industry (TCI) Chemicals (Tokyo, Japan); Presto Blue from Invitrogen (Carlsbad, CA, USA) and NucBlue are from Life Technologies (Carlsbad, CA, USA). A myeloperoxidase (MPO) assay kit was acquired from Abcam (Cambridge, UK), and the Lactate Dehydrogenase (LDH) assay kit was from Dojindo (Kumamoto, Japan). TNF-α, IL-1β and interleukin (IL)-6 proteins were bought from Sino Biological (Pennsylvania, USA). Lipopolysaccharide (LPS) was purchased from Sigma-Aldrich (St Louis, MO, USA). The Anti-NF-κB antibody, Anti-TNF-α antibody, Anti-IL-8 antibody, goat anti-rabbit IgG Alexa Fluor-488, and goat anti-mouse IgG Alexa Fluor-647 were purchased from Abcam.

### 2.1. Preparation of GAR-PLGA-ES100 NPs

The LIVINOL^TM^ (GAR)-PLGA-ES100 nanoparticles (GAR-PLGA-ES100 NPs) were prepared by adapting the solvent evaporation technique [23,24]. First, PLGA (20 mg) and GAR (5 mg) were dissolved in 1.5 mL of acetone. ES100 (20 mg) was also added to 1.5 mL of acetone, separately, for proper dissolving and then mixed with the above PLGA-GAR mixture. The organic phase, comprising PLGA-GAR-ES100, was then added to a 0.5% aqueous PVA solution, followed by sonication for 5 min at 40 kHz, and left to stir for 4 h (h). The evaporation of acetone assisted in the formation of NPs. The formed NPs were centrifuged for 30 min at 8000 rpm and at 4 °C, followed by subsequent washing with Milli Q water 4 times. The GAR-PLGA-ES100 NPs were lyophilized and stored at −20 °C for further studies, including characterization and in vitro cell studies. Coumarin-6-loaded fluorescent NPs were also prepared in the same way to study intracellular fate in CACO-2 cells.

### 2.2. Characterization of GAR-PLGA-ES100 NPs

#### 2.2.1. SEM

The size and surface morphology of GAR-PLGA-ES100 NPs were analyzed using SEM (HITACHI, SU-8030). The NPs (10 μL) dispersed in Milli Q water were drop-casted on clean silicon (Si) substrate, left for vacuum drying, and coated in Osmium for 20 s using a Neo Osmium Coater (Neoc-Pro coater). The coated sample was then viewed under SEM operated at an accelerating voltage of 5 kV.

#### 2.2.2. Size Distribution and Zeta Potential

To determine the average particle size and polydispersity index of the nanoparticles, dynamic light scattering using Zeta sizer (Malvern, Nano Zs) was utilized. The GAR-PLGA-ES100 NPs samples were suspended in Milli Q water and diluted before the measurements. The size distribution of the nanoparticles was analyzed using disposable sizing cuvettes. Zeta potential measurements were conducted using zeta dip cells. All measurements were measured in triplicates.

#### 2.2.3. Encapsulation Studies and Drug Loading Efficiency of GAR

The percentage encapsulation efficiency (% EE) of GAR inside GAR-PLGA-ES100 NPs was calculated using Equation (1):

(1) $$\%\;\mathrm{Encapsulation}\;\mathrm{Efficiency}=\frac{\mathrm{Initial}\;\mathrm{GAR}\;-\;\mathrm{Free}\;\mathrm{GAR}\;\mathrm{in}\;\mathrm{supernatant}}{\mathrm{Mass}\;\mathrm{of}\;\mathrm{initial}\;\mathrm{GAR}\;\mathrm{used}\;\mathrm{during}\;\mathrm{synthesis}}\;\times \;100$$

The calibration curve of the different concentrations of GAR in ethanol (5–50 μg/mL) versus absorbance was prepared. The absorbance peak of GAR (λ_MAX_ 356 nm) was measured using a UV-vis spectrophotometer (DU730 Beckman Coulter). The encapsulation efficiency was calculated by measuring the absorbance of the free GAR in the supernatant during the washing steps of NP preparation.

Drug loading efficiency was determined using Equation (2):

(2) $$\%\mathrm{Drug}\;\mathrm{Loading}\;=\frac{\mathrm{Weight}\;\mathrm{of}\;\mathrm{encapsulated}\;\mathrm{drug}}{\mathrm{Weight}\;\mathrm{of}\;\mathrm{GAR}-\mathrm{PLGA}-\mathrm{ES}100\;\mathrm{NPs}}\;\times \;100$$

#### 2.2.4. In Vitro Drug Release Studies

In vitro drug release studies of GAR from GAR-PLGA-ES100 NPs were carried out in PBS its different pH (1.2, 5.6, and 7.4) conditions. PLGA NPs loaded with GAR without ES100 coating (GAR-PLGA NPs) were also utilized in a comparative study to understand the effect of variation in pH, similar to the GIT environment necessitating the release of GAR into the medium. Approximately 2 mg of GAR-PLGA-ES100 NPs and GAR-PLGA NPs were dispersed in 1 mL of PBS containing 10% FBS with different pH, simulating the stomach (pH 1.2), small intestine (pH 5.6), and large intestine (pH 7.4). GAR-PLGA-ES100 NPs and GAR-PLGA NPs concentrations of 200 μg/mL were aliquoted in each tube from the 2 mg/mL stock solution. The samples were withdrawn periodically and centrifuged at a speed of 15,000 rpm for 30 min. The resultant supernatant was collected to measure the absorbance of the drug GAR (λ_MAX_ 356 nm) with a UV-vis spectrophotometer. The release profile of GAR was calculated with the following Equation (3):

(3) $$\%\;\mathrm{Release}\;\mathrm{of}\;\mathrm{GAR}=\frac{\mathrm{Released}\;\mathrm{GAR}}{\mathrm{Total}\;\mathrm{GAR}\;\mathrm{encapsulated}\;\mathrm{inside}\;\mathrm{NPs}}\;\times \;100$$

### 2.3. Cell Culture Maintenance

CACO-2 cells were purchased from the European Collection of Authenticated Cell Cultures (ECACC) and were grown using minimum essential medium (MEM) supplemented with non-essential amino acids (NEAA) and 10% FBS. CACO-2 cells have the functional properties of human intestinal cells generally used to screen intestinal permeability and drug absorption and study the active and passive diffusion transport of a drug and its carriers [25]. The cells were held in a humidified atmosphere with 5% CO_2_ at 37 °C until they reached 80–90% confluency. The cells were sub-cultured within 2–3 days and maintained in T25 flasks.

### 2.4. In Vitro Localization of Coumarin-6 PLGA-ES100 NPs

Fluorescent formulation of PLGA-ES100 NPs was obtained by the encapsulating a substitute fluorescent dye called Coumarin-6 in the NPs. The cellular uptake and distribution of NPs in CACO-2 cells were studied using confocal laser scanning microscopy (CLSM) (NIKON A1 plus). CACO-2 cells were seeded on confocal dishes (4 × 10^3^ cells) and grown until they formed a confluent monolayer at 37 °C in a humidified atmosphere. Then, they were incubated with coumarin-6 PLGA-ES100 NPs in a 50 μg/mL concentration for 4 h at 37 °C in a humidified atmosphere. After 4 h of incubation, the culture media of the NPs was aspirated and washed with 500 μL of PBS for 2 min at least 3 times. The cells were then stained with a nuclear dye Nuc Blue Live Ready probes for an additional 20–30 min at room temperature. Control cells were also stained with Nuc Blue Live Ready probes. The fluorescence of coumarin-6-loaded NPs in the cells is observed at an excitation/emission range of 488 nm/525 nm under a fluorescein isothiocyanate (FITC) laser filter channel. Nuc Blue has an excitation/emission range of 360 nm/460 nm.

### 2.5. In Vitro Cytotoxicity Analysis of GAR-PLGA-ES100 NPs in CACO-2 Cells

Cytotoxicity analysis of GAR-PLGA-ES100 NPs, blank-PLGA-ES100 NPs, and free GAR at different concentrations was conducted on CACO-2 cells to study therapeutic efficacy and biocompatibility of each of them. Presto Blue fluorometric assay was utilized for this experiment. The cells were seeded into 96-well microtiter plates at plating densities of 5–6 × 10^3^ cells /mL and grown until they reached 70–80% confluency for the experiment. GAR-PLGA-ES100 NPs were added to the CACO-2 cells at different concentrations of 50, 100, 250, and 500 μg/mL. Free GAR was added at the 50 μg/mL concentration after optimization, and blank-PLGA-ES100 NPs was added at 500 μg/mL. After adding the respective NPs and free GAR, the cells were incubated for a 24-48-h treatment period. At the end of each treatment period, the NPs were removed, followed by fresh media, and 10 μL of Presto Blue dye was added to each well. The cells were incubated for 2–3 h at 37 °C, and the fluorescence was recorded at 560 nm Ex/590 nm Em using a microplate reader (Spectra max i3x, Multimode microplate reader, Molecular Devices). Untreated cells were taken as controls.

### 2.6. Lactate Dehydrogenase (LDH) Release Assay

The inflammatory damage induced by LPS in CACO-2 cells in the absence or presence of blank-PLGA-ES100 NPs (500 μg/mL), GAR-PLGA-ES100 NPs (50–500 μg/mL), and free GAR (50 μg/mL) was studied using lactate dehydrogenase (LDH) release assay with a commercial detection kit (Dojindo, Japan) and was applied according to the manufacturer’s instructions. LPS is a main component of the outer membrane of Gram-negative bacteria, can induce inflammation in the gut and is a significant risk factor of IBD [26]. The cells were seeded at 5 × 10^5^ cells/well in 96 well plates and incubated at 37 °C with 5% CO_2_ to reach confluency. Once confluent, the cells were initially induced with LPS at 20 μg/mL for 24 h, followed by the addition of fresh media with blank-PLGA-ES100 NPs (500 μg/mL), GAR-PLGA-ES100 NPs (50–250 μg/mL), and free GAR (50 μg/mL) for 24 h of treatment. Absorption was measured at 490 nm by utilizing a Spectra max i3x Multimode microplate reader (Molecular Devices).

### 2.7. Myeloperoxidase (MPO) Assay

An MPO assay has been performed to quantify the inflammation reduced by the GAR-PLGA-ES100 NPs in the inflamed cells [27]. The MPO assay was conducted according to the manufacturer’s instructions (Abcam, United Kingdom). Initially, the cells were seeded at 1 × 10^5^ cells/well in 12 well plates and allowed to grow until they reached confluency. The cells were pre-treated with LPS (20 μg/mL) in fresh media for 24 h. They were washed the next day with PBS buffer, followed by the addition of blank-PLGA-ES100 NPs (500 μg/mL), GAR-PLGA-ES100 NPs (50–500 μg/mL), and free GAR (50 μg/mL) for 24 h of treatment. The next day, the NPs and free drug were aspirated, and the cells were collected to obtain the supernatants for the MPO activity assessment. The fluorescence was measured at E*x*/E*m* = 484/525 nm on a microplate reader (Spectra max i3x, Multimode microplate reader, Molecular Devices).

### 2.8. Induction of Inflammation

CACO-2 cells were seeded at 4 × 10^3^ cells/well until confluent in a glass-bottom dish to induce inflammation. Once confluent, the cells were divided into sections to study the expression of different inflammatory cytokines (TNF-α), chemokines (IL-8), and NF-κB. The cells were treated with a specific concentration of the pro-inflammatory cocktail (PIC) for each protein of interest and for a specific time to understand and analyze the induction of inflammation in CACO-2 cells (Table 1).

After the incubation period, the cells were washed with PBS thrice and fixed in 4% paraformaldehyde for 20 min at room temperature. The cells were rinsed briefly with PBS thrice and then permeabilized in cold methanol for 5–10 min at −20 °C. After the additional washing, cells were incubated with primary antibodies against TNF-α (1:200), IL-8 (1:200), and NF-κB (1:200) overnight at 4 °C. The next day, cells treated with primary antibodies against TNF-α, IL-8, and NF-κB were labelled with goat anti-rabbit IgG Alexa Fluor-488 conjugated secondary for 45–60 min at room temperature. All the cells were then washed with PBS and incubated with Nuc Blue Live Ready Probes. The cells were observed and imaged under CLSM (Nikon A1plus). Cells without the cocktail treatment were also used to differentiate between non-inflamed and inflamed state.

### 2.9. Inhibition of Inflammation

After we studied the induction of inflammation using PIC, the next step involved investigating the anti-inflammatory effect of GAR-PLGA-ES100 NPs in the inflamed CACO-2 cells. CACO-2 cells treated with PIC in their respective concentrations (Table 1) for inducing the expression of NF-κB, TNF-α, and IL-8 were incubated at 37 °C. Later, the inflamed CACO-2 cells were washed with PBS, and subsequently, GAR-PLGA-ES100 NPs (250 μg/mL) in fresh media (500 μL) was added to the cells for 48 h and incubated at 37 °C. After treatment with GAR-PLGA-ES100 NPs, the immunofluorescence assay to detect reduction in the expression of NF-κB, TNF-α, and IL-8 in CACO-2 cells was prepared as mentioned above in the previous step.

### 2.10. Statistical Analysis

The data were expressed as the data mean ± standard error (S.E.M). The significance of difference was analyzed by unpaired *t*-test. The analysis was also carried out using GraphPad Prism. The data were considered significant when *p* < 0.05.

## 3. Results

### 3.1. Synthesis and Characterization of GAR-PLGA-ES100 NPs

**Figure 1:**
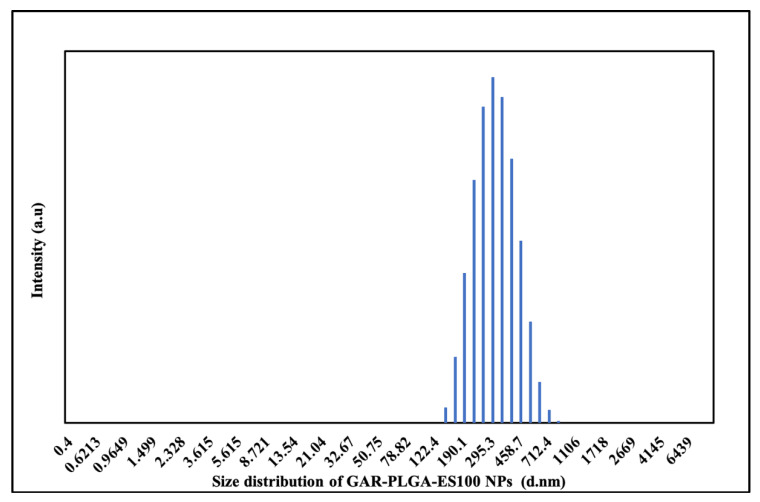
Size distribution of GAR-PLGA-ES100 NPs by DLS measurement.

**Figure 2:**
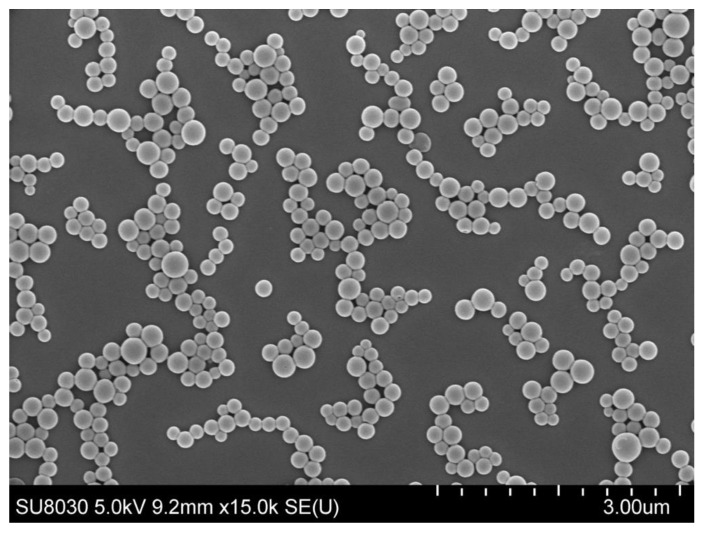
SEM image of GAR-PLGA-ES100 NPs (At scale 3.00 μm).

The preparation of the GAR-PLGA-ES100 NPs was carried out via solvent evaporation technique. The particle size and PDI were measured using dynamic light scattering DLS with a Zeta sizer (Malvern, Nano Zs) (Figure 1). According to the analyses, the particle size and PDI were 295 nm and 0.1, respectively, for GAR-PLGA-ES100 NPs. The SEM images of the nanoparticles were also in correlation with the DLS data. The size, shape, and morphology of the GAR-PLGA-ES100 NPs were found to be spherical and smooth-surfaced, as observed in SEM analysis (Figure 2). The zeta potential of the GAR-PLGA-ES100 NPs was −23.1 mV. The amount of GAR encapsulated by the PLGA polymer coated with ES100 was calculated using the spectrophotometric method. The % EE and % drug-loading efficiency of GAR in the NPs were 91.2% and 45.6%, respectively.

### 3.2. In Vitro Drug Release

**Figure 3:**
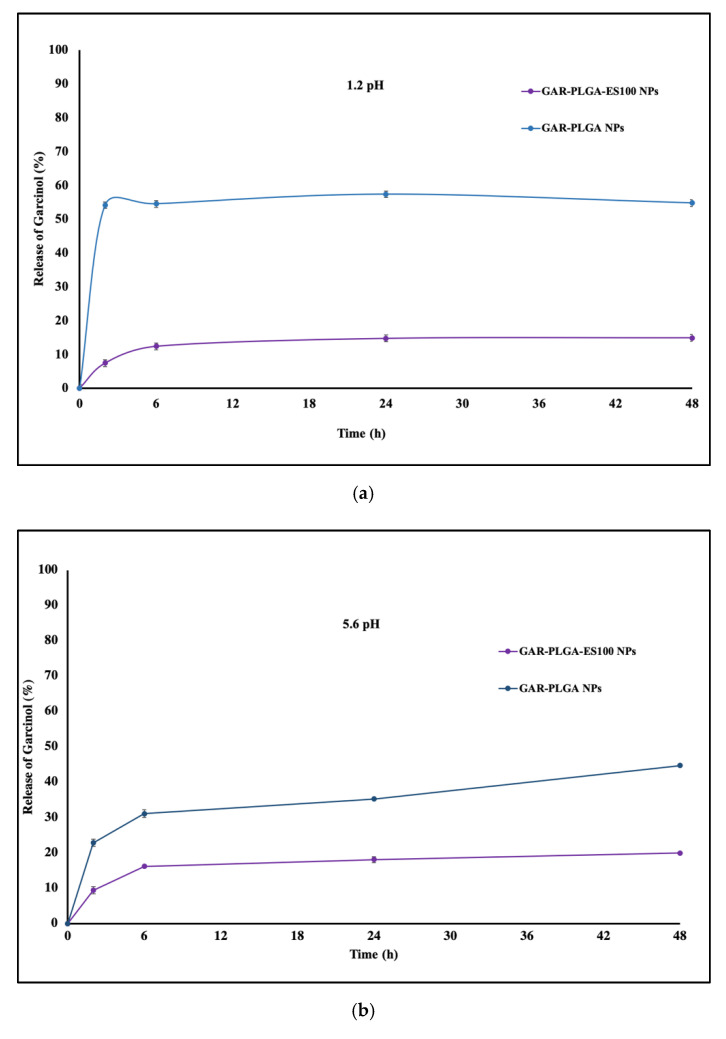

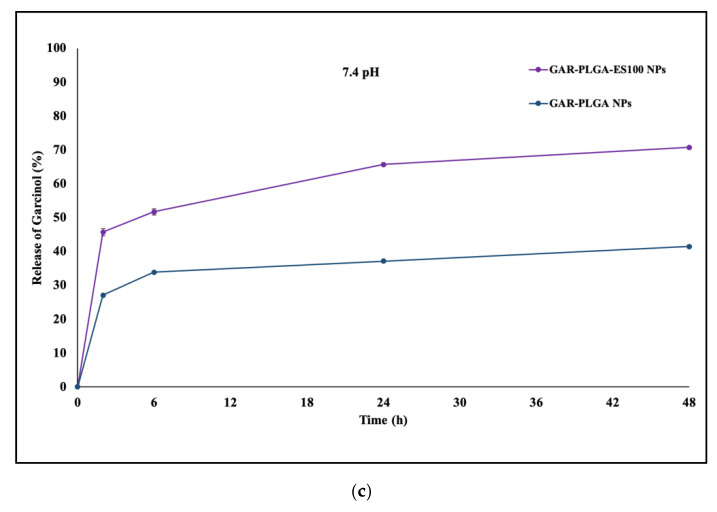
(**a**) In vitro release of GAR from GAR-PLGA-ES100 NPs and GAR-PLGA NPs in phosphate buffered saline (PBS) (pH 1.2, PBS/FBS buffer) studied over 2 days at 37 °C. (**b**): In vitro release of GAR from GAR-PLGA-ES100 NPs and GAR-PLGA NPs in PBS (pH 5.6, PBS/FBS buffer) studied over 2 days at 37 °C. (**c**): In vitro release of GAR from GAR-PLGA-ES100 NPs and GAR-PLGA NPs in PBS (pH 7.4 PBS/FBS buffer) studied over 2 days at 37 °C.

The in vitro release of GAR by GAR-PLGA-ES100 NPs and GAR-PLGA NPs in PBS with 10% FBS (pH 1.2, 5.6, and 7.4) are shown in Figure 3a–c. The drug release from the respective NPs was studied after of 2, 6, 24, and 48 h. The initial burst release of GAR from GAR-PLGA NPs was seen to be 54.12 ± 0.33% in pH 1.2, which continued up to 48 h, retaining the same percentage drug release. However, GAR-PLGA-ES100 NPs showed that only 7.39 ± 0.63% of the drug was released in the initial 2 h at pH 1.2. Later, as it progressed to 24 and 48 h, the drug release was only double the initial burst with 9.98 ± 0.12% and 20.12 ±  0.37%, respectively. At pH 5.6, GAR-PLGA NPs showed an initial release of 22.8 ± 1.16%, whereas GAR-PLGA-ES100 NPs had only a 9.44 ± 0.186% release of the drug. As time proceeded, at 48 h of the study, around 44.78 ± 0.33% of the drug was released by GAR-PLGA NPs and only 20% of the drug was released in GAR-PLGA-ES100 NPs. At pH 7.4, which resembles the neutral pH of the colon, the GAR-PLGA NPs only released 27.1 ± 0.06% of the drug within 2 h. The GAR-PLGA-ES100 NPs demonstrated a 45.69 ± 0.9% release, followed by a 70.2 ± 0.433% release at 48 h. Among all the tested pH, we found GAR from GAR-PLGA-ES100 NPs were released the most at pH 7.4 compared with pH 1.2 and pH 5.6.

### 3.3. Localization of the NPs in CACO-2 Cells

**Figure 4:**
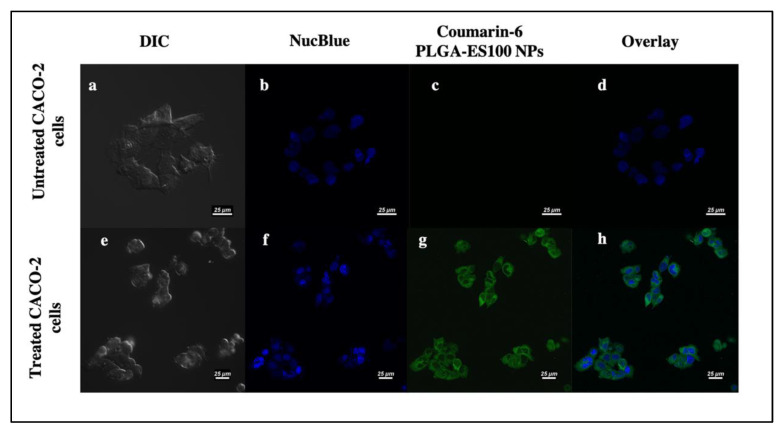
Cellular uptake of Coumarin-6 PLGA-ES100 NPs in CACO-2 cells by confocal microscopy; untreated CACO-2 cells (**a**–**d**), CACO-2 cells treated with Coumarin-6 PLGA-ES100 NPs for 4 h (**e**–**h**) (on a scale of 25 μm).

The cellular uptake of GAR could be studied using a substitute fluorescent dye encapsulated inside PLGA-ES100 NPs. In our study, we have used Coumarin-6-loaded PLGA-ES100 NPs to monitor the cellular fate of the NPs. As shown in Figure 4, the coumarin-6-loaded NPs were readily internalized within the cytoplasm of the cells, which could be due to the endocytic process [28].

### 3.4. In Vitro Cytotoxicity Analysis

**Figure 5:**
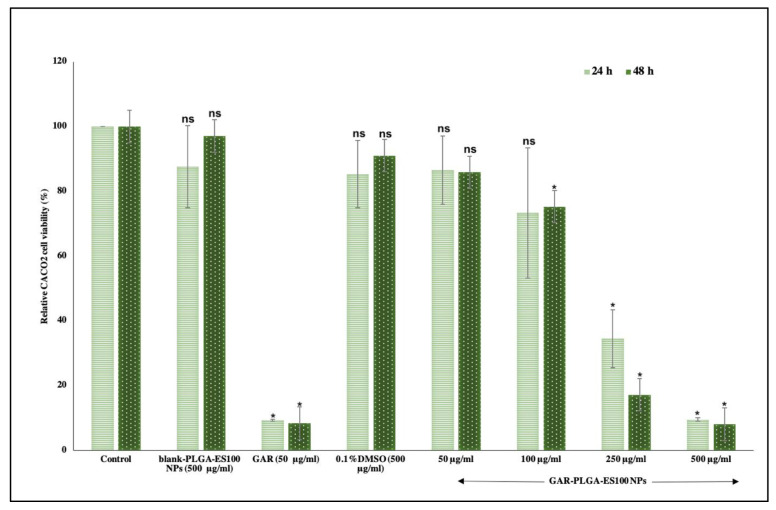
In vitro cytotoxicity assay on CACO-2 cells for 24–48 h. blank-PLGA-ES100 NPs concentration was 500 μg/mL, free GAR was 50 μg/mL and GAR-PLGA-ES100 NPs (50, 100, 250, and 500 μg/mL). Student’s unpaired t-test was carried out to check the statistical significance of the experiment (ns: not significant; * *p* < 0.05).

In vitro cytotoxicity analysis is conducted to check the biocompatibility of the GAR-PLGA-ES100 NPs in CACO-2 cells using Presto blue assay (Figure 5). Blank-PLGA-ES100 NPs only constituting of polymers did not seem to affect the viability of the cells at 500 μg/mL (the maximum concentration) for 24 and 48 h. The GAR-PLGA-ES100 NPs were least toxic at concentrations of 50 and 100 μg/mL. However, at 250 μg/mL, the GAR-PLGA-ES100 NPs were slightly cytotoxic and highly cytotoxic at a concentration of 500 μg/mL at 24 and 48 h of incubation. Free GAR was found to be cytotoxic to CACO-2 cells, and cell viability was drastically reduced to 9.26% ± 0.26 and 8.3% ± 0.24 during 24 and 48 h of incubation, respectively.

### 3.5. LDH Activity

**Figure 6:**
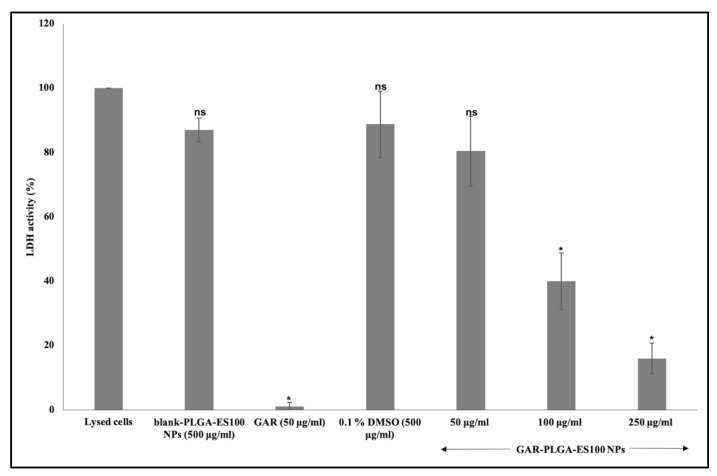
Lactate dehydrogenase (LDH) assay on CACO-2 cells treated for 24 h with blank-PLGA-ES100 NPs. Concentration was 500 μg/mL, free GAR was 50 μg/mL and GAR-PLGA-ES100 NPs (50, 100, and 250 μg/mL). Student’s unpaired t-test was carried out to check the statistical significance of the experiment (ns: not significant; * *p* < 0.05).

The reduction of inflammatory damages on CACO-2 with different concentrations of GAR-PLGA-ES100 NPs was also carried out using LDH assay. The LDH is an enzyme localized in the cytosol and released by damaged cell [5]. In this study, we analyzed LDH activity using blank-PLGA-ES100 NPs, GAR-PLGA-ES100 NPs, and free GAR (Figure 6). It is demonstrated that the LDH activity in blank-PLGA-ES100 NPs treated on LPS-induced inflamed CACO-2 cells was 86.96% ± 3.68. However, with the treatment of GAR-PLGA-ES100 NPs on LPS-induced inflamed CACO-2 cells, LDH activity reduced tremendously. The concentration of 100 μg/mL decreased LDH activity to 40.04%  ± 8.8, and 250 μg/mL reduced the LDH activity to 15.99%  ± 4.78. Free GAR reduced the LDH activity to 1.04% ± 2.74.

### 3.6. MPO Activity

**Figure 7:**
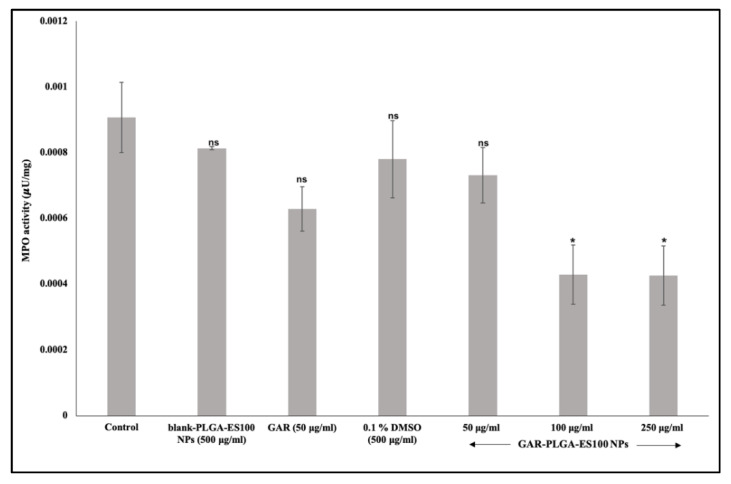
Myeloperoxidase (MPO) assay on CACO-2 cells treated for 24 h with blank-PLGA-ES100 NPs. Concentration was 500 μg/mL, free GAR was 50 μg/mL and GAR-PLGA-ES100 NPs (50, 100, and 250 μg/mL). Student’s unpaired t-test was carried out to check the statistical significance of the experiment (ns: not significant; * *p* < 0.05).

This study noticed that MPO activity in the LPS-induced inflamed CACO-2 control cells was elevated compared with the GAR-PLGA-ES100 NP-treated CACO-2 cells (Figure 7). After the treatment, GAR-PLGA-ES100 NPs (50–250 μg/mL) were seen to be more effective than free GAR (50 μg/mL) in reducing MPO activity in inflamed CACO-2 cells. However, the blank-PLGA-ES100 NPs didn’t seem to entirely reduce MPO activity due to the absence of any anti-inflammatory property by itself. Collectively, these results confirm the application of the anti-inflammatory properties of GAR-PLGA-ES100 NPs in the treatment of IBD.

### 3.7. Induction and Inhibition of Inflammation Using Immunofluorescence Assay

**Figure 8:**
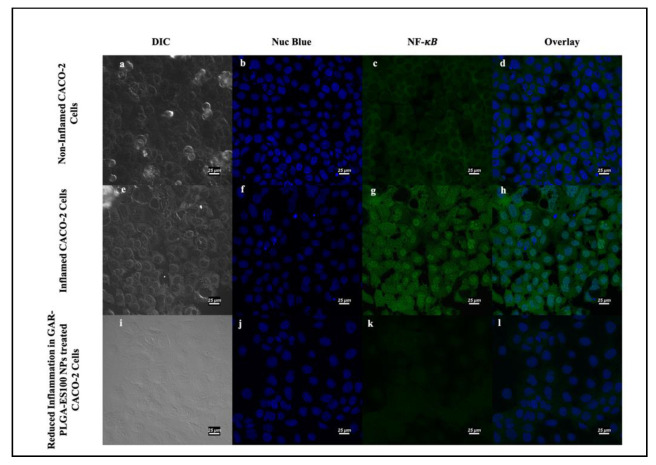
Induction and inhibition of inflammation in CACO-2 cells by confocal microscopy: non-inflamed CACO2 cells (**a**–**d**); inflamed CACO-2 cells treated with proinflammatory cocktail comprising of IL-6, TNF-α, and LPS at 0.2, 0.3, and 20 μg/mL, respectively, for 3 h used to observe the expression of NF-κB (**e**–**h**). Reduction in the expression of NF-κB occured in CACO-2 cells after treatment with GAR-PLGA-ES100 NPs (250 μg/mL) for 48 h (**i**–**l**) (at scale 25 μm).

We have examined the expression of NF-κB, TNF-α, and IL-8 stimulated by PIC in CACO-2 cells using immunofluorescence assay. As seen in Figure 8a–d, NF-κB expression was seen without any addition of pro-inflammatory cytokines in the non-inflamed cells [29]. When there is deregulated inflammation manually induced via treatment with PIC (Figure 8e–h), it can cause excessive and long-lasting damage, leading to the development of IBD [30]. The inflamed CACO-2 cells, when treated with GAR-PLGA-ES100 NPs (250 μg/mL), were able to reduce the expression of NF-κB within 48 h of treatment (Figure 8i–l).

**Figure 9:**
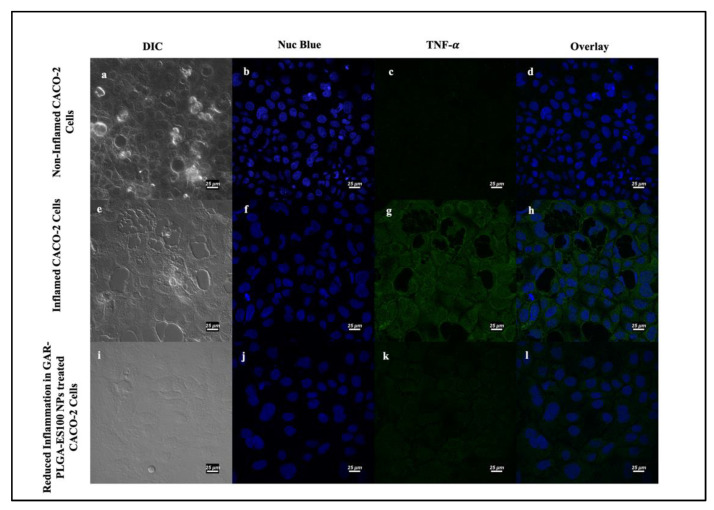
Induction and inhibition of inflammation in CACO-2 cells by confocal microscopy: non-inflamed CACO2 cells (**a**–**d**); inflamed CACO-2 cells treated with proinflammatory cocktail comprising of IL-6, TNF-α, and LPS at 0.4, 0.9, and 30 μg/mL, respectively, for 3 h used to observe the expression of TNF- α (**e**–**h**). Reduction in the expression of TNF- α occurred in CACO-2 cells after treatment with GAR-PLGA-ES100 NPs (250 μg/mL) for 48 h (**i**–**l**) (at scale 25 μm).

**Figure 10:**
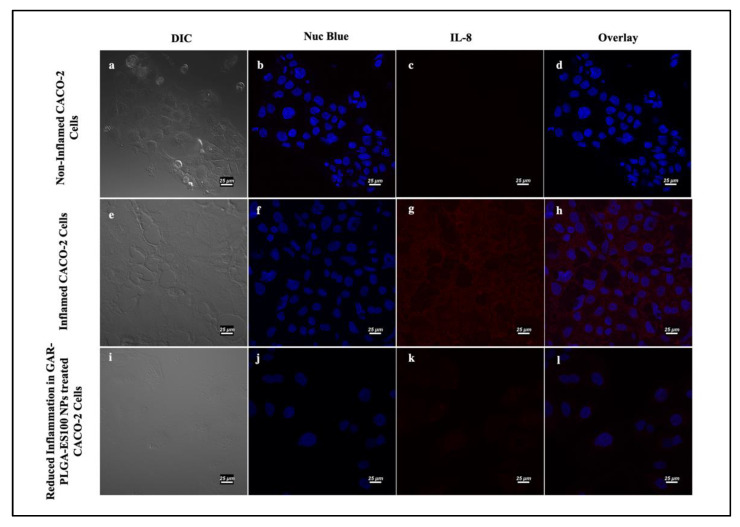
Induction and inhibition of inflammation in CACO-2 cells by confocal microscopy: non-inflamed CACO2 cells (**a**–**d**); CACO-2 cells treated with proinflammatory cocktail comprising of IL-6, TNF-α, IL-1β, and LPS at 0.4, 0.9, 1, and 30 μg/mL, respectively, for 45 min demonstrated the expression of IL-8 (**e**–**h**). Reduction in the expression of IL-8 in CACO-2 cells was observed after treatment with GAR-PLGA-ES100 NPs (250 μg/mL) for 48 h (**i**–**l**) (at scale 25 μm).

Next, we investigated the TNF-α and IL-8 levels’ expression without any addition of PIC, as seen in Figure 9a–d and Figure 10a–d, revealing no respective expressions of the proteins. As soon as PIC was manually introduced into the cells to induce inflammation, TNF-α and IL-8 levels increased, as provided in Figure 9e–h and Figure 10e–h, respectively. After the treatment with GAR-PLGA-ES100 NPs, the expression of TNF-α (Figure 9i–l) and IL-8 (Figure 10i–l) was extensively reduced within 48 h of treatment.

## 4. Discussion

Improving the bioavailability of anti-inflammatory agents, anti-oxidants and anti-cancer agents is a crucial task; therefore, incorporating them into nanocarriers that are small in size, have an increased surface area, are high reactivity, and show stability can promote GAR as a highly effective drug [31]. During the synthesis of the NPs using the solvent evaporation technique, various parameters were assessed, including polymer content, surfactant concentration, sonication, and stirring process for optimization [32]. The different concentrations of polymers selected for the study were 20, 40 and 50 mg. The formation of larger NPs, 300–400 nm in size, occurred with aggregation when the polymer concentrations increased. The concentration of surfactant (0.1%, 0.3% and 0.5%) also influenced the formation of NPs where an increase in the concentrations of PVA showed a reduction in the size of the NPs to the desired size range spherical morphology. Another important parameter was the sonication (1, 2, 3 and 5 min) and stirring time (1, 2, and 4 h). Extension of sonication and stirring time showed a decrease in the size of the NPs as well. Therefore, based on these parameters, we found that at a polymer concentration of 20 mg, a PVA concentration of 0.5%, sonication for 2 min and magnetic stirring for 4 h, the fabricated NPs reached the size and shape appropriate for inflamed colonic uptake [33]. The negative surface charge of the GAR-PLGA-ES100 NPs also protects the NPs from not adhering to the mucosal layer due to electrostatic repulsion and can release the drug in a sustained manner at the site of inflammation [34]. Hence, the nanometer scale size of GAR-PLGA-ES100 NPs, along with the negative charge and high encapsulation, makes them favorable candidates for IBD treatment.

The GAR-PLGA-ES100 NPs were able to withstand the drug release in acidic pH 1.2 and 5.6, compared with the uncoated GAR-PLGA NPs, due to the active presence of ES100 [35] as a surface coating. The initial burst of the drugs from the NPs could be due to the loosely bound drug present on the surface of the polymer during fabrication of the NPs [19]. It was also revealed that when the NPs reached pH 7.0, there was a sustained pattern of drug release [36]. The coating of NPs with ES100 enables the drug to be released at the colon’s pH (pH ≥ 7) [37]. This phenomenon is explained by the deprotonation of the carboxylic functional groups of the ES100 polymer at the pH levels of the colon, where ES100 dissolves and swells, leading to the release of the entrapped GAR from the pH-sensitive NPs [23]. The formulation of pH-sensitive NPs using PLGA and Eudragit^®^ loaded with budesonide (BSD) also showed sustained release of the drug at the colonic pH and increased more therapeutic effects than BSD alone when treated on a TNBS-induced animal model of colitis [38]. The trans-cellular movement of NPs across intestinal barriers depends on the shape, size, surface chemistry, and charge [39]. Coumarin-6-loaded NPs helped trace their intracellular fate in the CACO-2 cell monolayer. Previous studies have also shown the cellular uptake of coumarin-6-loaded NPs within 4 h of incubation in CACO-2 cells [40]. The cytotoxicity of GAR on CACO-2 studies for IBD has not been recently studied, but it was shown that other cancer cell lines related to the colon, like HCT-116 and HT29, were found to be very sensitive to the inhibitory effect of GAR when compared with normal cells [41].

The in vitro cytotoxic experiment performed on CACO-2 cells demonstrated that the selection of GAR-PLGA-ES100 NPs concentrations were less cytotoxic than free GAR. The lower concentration of GAR-PLGA-ES100 NPs (50 μg/mL) was found to be cytocompatible with more than 80% cell viability. The concentrations of 100 μg/mL maintained up to 70% cell viability. However, the concentration of 250 μg/mL was found to be cytotoxic with less than 50% cell viability. Finally, the highest concentration, 500 μg/mL, was extremely cytotoxic with less than 10% cell viability. Compared to free GAR toxicity, the NPs tend to show a sustained reduction of cell viability to the CACO-2 cells, which is advantageous to minimize severe side-effects and selective targeting. The mechanism of inhibition of inflammation was further assured by LDH and MPO assays. The anti-inflammatory effect of GAR-PLGA-ES100 NPs was evaluated in vitro in LPS-activated inflammation in CACO-2 cells. LPS can alter the intestinal epithelia’s barrier function, resulting in increased permeability or disrupted tight junctions [26]. The LDH activity of the LPS-induced inflamed CACO-2 cells was found to be highly elevated. In this study, we observed that GAR-PLGA-ES100 NPs were able to inhibit LPS-induced cell damage by reducing inflammation. The blank-PLGA-ES100 NPs were found to be incapable of reducing LDH activity since they lacked anti-inflammatory properties by themselves. R. Coco et al. [33] conducted a study on the cytotoxicity of different nano-formulations using PLGA and ES100 in CACO-2 cells and concluded that the LDH activity reduced to 15%. Even though free GAR is also capable of reducing LDH activity [42], being a hydrophobic drug, aqueous insolubility is a barrier to delivering it in solution form or achieving high bioavailability [43]. However, GAR-PLGA-ES100 NPs (250 μg/mL) was shown to elicit a tremendous reduction in LDH activity. Similarly, we analyzed the reduction of MPO activity in GAR-PLGA-ES100 NP-treated CACO-2 cells compared with free GAR. Myeloperoxidase is considered a biomarker of inflammation since the enzyme is released into the extracellular medium during the inflammatory process [44]. It catalyzes the formation of sodium hypochlorite acid from hydrogen peroxide and sodium chloride [45]. *Garcinia combogia* extract, previously used to study its effect in reducing MPO activity in colitis induced in rats as an oral delivery method, has shown progressive results [46]. The current study observed that in GAR-PLGA-ES100 NP-treated (250 μg/mL) CACO-2 cells showed reduced MPO activity compared with CACO-2 cells treated with free GAR. Thus, this demonstrates GAR-PLGA-ES100 NPs to be a beneficial factor in producing anti-inflammatory effects for IBD treatment.

Since the 250 μg/mL concentration of GAR-PLGA-ES100 NPs induced reduction in both LDH and MPO activity in CACO-2 cells, it has been decided to incorporate this concentration in further studies involving the expression of NF-κB, TNF-α, and IL-8. In another study, the nuclear translocation of the p65 subunit belonging to NF-κB was also observed using immunofluorescence assay on CACO-2 cells treated with LPS [26]. The study, conducted by Stevens et al., [47] has determined that IL-6, found only in IBD specimens, as well as IL-1β, and TNF-α promote local inflammation. These signaling pathways also appear to have distinct roles in the secretion of IL-8 in response to different antagonists [48]. The activation of NF-κB in intestinal epithelial cells (IEC) can increase IEC-derived inflammatory cytokines, including TNF-α and IL-8 [49]. In the present study, we observed the anti-inflammatory effect of GAR-PLGA-ES100 NPs on PIC-induced inflammation in CACO-2 cells, indicating that GAR-PLGA-ES100 NPs possesses anti-inflammatory potential in inflamed IECs. The introduction of GAR into anti-inflammatory therapy has started recently, although the factors behind its capabilities are still yet to be discovered [43]. GAR has previously shown to have reduced inflammatory markers like TNF-α and NF-κB in hepatic inflammation in the steatohepatitis-derived hepatocellular carcinoma of a mouse model (STAM) and LPS-induced inflammation in THP-1 cells in vitro [50]. The inhibition of inflammation by GAR is expected due to its ability to down-regulate NF-κB and cyclooxygenase-2 (COX-2) [51]. This inhibition of NF-κB to the DNA also can down-regulate a number of its target genes involved in the process [52]. Hence, we observed a reduction in TNF-α and IL-8 secretions in the confocal images.

## 5. Conclusions

The inflamed colon is a problematic area for targeted drug delivery due to general considerations of the gastrointestinal tract. Conventional treatment methods include additional burdens of adverse toxicity and systemic side effects. Different colonic approaches can be used to tackle these issues. In conclusion, we have formulated pH-sensitive and biodegradable NPs for oral drug delivery in IBD treatment. The GAR-PLGA-ES100 NPs were found to be 295 nm in size with a smooth surface. Due to ES100 coating, GAR-PLGA-ES100 NPs were able to withstand drug release at an acidic pH resembling the stomach and small intestine. The in vitro drug release revealed that NPs only released the drug at the pH simulating the colon (pH > 7). The relative CACO-2 cell viability demonstrated that the NPs were the less toxic compared with free GAR. Fluorescent NPs were internalized by the CACO-2 cells upon 4 h of incubation. The reduction of the inflammation of the NPs using LDH assay proved to be effective, and GAR-PLGA-ES100 NPs were able to reduce LPS-induced inflammation by reducing MPO activity. The inhibitory effect of GAR-PLGA-ES100 NPs on the expression of NF-κB, TNF-α, and IL-8 induced by PIC demonstrates the benefit of these NPs in IBD treatment. 

## Figures and Tables

**Table 1:** Pro-Inflammatory Cytokines Cocktail (PIC) Concentrations Added for the Induction of Inflammation in CACO-2 Cells to Study the Expression of Nuclear Factor Kappa B cell (NF-κB), Tumor Necrosis Factor (TNF)-α, and Interleukin (IL)-8.

Protein of Interest	IL-6	TNF-α	LPS	IL-1β	Time
**TNF-** α	0.4 (μg/mL)	0.9 (μg/mL)	30 (μg/mL)	-	3 h
**NF-** κ **B**	0.2 (μg/mL)	0.3 (μg/mL)	20 (μg/mL)	-	3 h
**IL-8**	0.4 (μg/mL)	0.9 (μg/mL)	30 (μg/mL)	1 (μg/mL)	45 min

## Data Availability

Data sharing not applicable.
